# Does Sleep Play a Role in Memory Consolidation? A Comparative Test

**DOI:** 10.1371/journal.pone.0004609

**Published:** 2009-02-25

**Authors:** Isabella Capellini, Patrick McNamara, Brian T. Preston, Charles L. Nunn, Robert A. Barton

**Affiliations:** 1 Department of Anthropology, Durham University, Durham, United Kingdom; 2 Department of Neurology, Boston University School of Medicine and Boston VA Healthcare System, Boston, Massachusetts, United States of America; 3 Department of Anthropology, Harvard University, Cambridge, Massachusetts, United States of America; 4 Max Planck Institute for Evolutionary Anthropology, Leipzig, Germany; 5 Department of Integrative Biology, University of California, Berkeley, California, United States of America; Indiana University, United States of America

## Abstract

Sleep is a pervasive characteristic of mammalian species, yet its purpose remains obscure. It is often proposed that ‘sleep is for the brain’, a view that is supported by experimental studies showing that sleep improves cognitive processes such as memory consolidation. Some comparative studies have also reported that mammalian sleep durations are higher among more encephalized species. However, no study has assessed the relationship between sleep and the brain structures that are implicated in specific cognitive processes across species. The hippocampus, neocortex and amygdala are important for memory consolidation and learning and are also in a highly actived state during sleep. We therefore investigated the evolutionary relationship between mammalian sleep and the size of these brain structures using phylogenetic comparative methods. We found that evolutionary increases in the size of the amygdala are associated with corresponding increases in NREM sleep durations. These results are consistent with the hypothesis that NREM sleep is functionally linked with specializations of the amygdala, including perhaps memory processing.

## Introduction

It has been suggested that sleep is of particular importance to brain processes such as memory consolidation and learning [1]–[4]. Experimental studies have supported this ‘memory consolidation’ hypothesis of sleep function by showing that sleep-deprived human and animal subjects perform poorly in learning tasks when compared to individuals that are well rested [2], [5]. However, the approach and conclusions of these studies are often criticized, due to the stress associated with sleep deprivation experiments [6], [7] and because memory consolidation can also occur in the absence of sleep.

The comparative study of sleep variation offers a complementary approach to investigating potential adaptive functions of sleep [8], [9] and most comparative research has focused on sleep durations. The importance of sleep times is reflected by the observation that when sleep deprived, experimental human and animal subjects exhibit a ‘sleep rebound’ proportional to the amount of sleep lost [10], indicating that the amount of sleep, or of some specific component of sleep, is physiologically relevant. Previous comparative studies have suggested that the great interspecific variation in sleep durations observed in mammals may reflect either functional benefits or ecological constraints, or both [e.g. 9],[11],[12]–[15]. Recent analyses on mammalian sleep durations have reported a positive relationship between rapid-eye-movement (REM) sleep and mammalian whole brain volume, which has been taken as support for a cognitive function of sleep [8], [12] [but see 11]. While these reports are consistent with a memory related function of sleep, the brain is a complex organ, and comparative evidence suggests that functionally specific regions have changed in size independently of whole brain size [16]. Measures of total brain size or of encephalization are therefore too coarse to substantiate the idea of a functional association between sleep and specific cognitive processes. A potentially more targeted approach is to examine sleep parameters in relation to specific brain regions that play a role in memory consolidation.

Ecologically-imposed needs for increased memory capacity should be reflected by an increase in the size of brain structures that are responsible for memory processing and consolidation [17]. For example, spatial memory is important in animals that hoard food because it improves their ability to retrieve stored food at a later time, which in turn enhances fitness. The hippocampus is one of the most important brain structures involved in spatial memory processing and memory retention, and studies in birds have shown that hippocampal volumes and hippocampal neuron numbers are higher in species and populations that cache food relative to those that do not exhibit such behaviour [17]–[19]. Similarly, if sleep serves a specific function with regard to memory consolidation and learning, we expect that greater memory-related demands result in a greater need for sleep. Those brain structures that are devoted to memory processing and learning therefore should be positively associated with sleep durations.

Many brain regions are involved in the diverse aspects of memory formation, but those hypothesised to have prominent roles in forming adaptively relevant associations in mammals include the hippocampus, amygdala and neocortex [17], [20]–[24]. During sleep a variety of brain structures are in a highly activated state, including those specifically linked to memory consolidation and learning. Interactions of the amygdala and hippocampus with one another and the neocortex during NREM sleep are well documented [25], while the outflow from the hippocampus to the neocortex is inhibited during REM but not NREM sleep [22], [25]. Recent studies converge on the conclusion that procedural and emotional forms of memory benefit from both REM and NREM sleep, while episodic memory benefits only from NREM sleep [4], [26]–[28].

Here we test the hypothesis that sleep is involved in memory consolidation and learning processes, predicting that evolutionary increases in the relative size of mammalian neocortical, hippocampal, and amygdalar regions will be associated with increased durations of REM and NREM sleep. We test these predictions by conducting phylogenetically controlled analyses of mammalian sleep durations and brain structures.

## Materials and Methods

We constructed a dataset of mammalian sleep durations (REM and NREM sleep times in hours/day) from an exhaustive search of the published literature [29; data available at http://www.bu.edu/phylogeny/index.html]. In previous analyses of this dataset we found that when sleep was recorded for less than 12 hours, sleep times were significantly underestimated, and that EEG studies tended to have lower estimates of sleep durations relative to non-EEG behavioural studies [11]. We thus restricted our analyses to studies that recorded sleep durations with EEG equipment for at least 12 hours. We excluded monotremes and aquatic mammals because their peculiar sleep architecture may not be comparable to that of terrestrial mammals [9], [30].

We extracted data on overall brain volume and the total volumes of individual brain components from a paper that employed uniform measurement procedures across species [31]. Data on neocortical and hippocampal volumes were available for 14 species in our sleep dataset, and data on amygdalar volumes for 13 species. Our final dataset comprised eight primates, one tree shrew, three ‘insectivores’, and two rodents (see Appendix S1). All variables were log-transformed to achieve normality.

Because closely related species tend to exhibit similar sleep durations [11], we implemented statistical methods that explicitly incorporate phylogeny to account for the lack of statistical independence in the data due to common ancestry [32]–[35]. Specifically we used the program BayesTraits [36], [37] to perform a multiple regression analysis with the method of phylogenetic generalized least squares (PGLS). PGLS converts the phylogeny into a variance-covariance matrix of species relationships, which is then used to weight the parameters of regression analysis estimated with maximum likelihood [ML, 36], [37]. We based our tests on the mammalian phylogenetic tree by Bininda-Emonds et al. [38] with updated branch lengths [39].

For each brain structure volume we calculated the volume of the remaining brain as a log-transformed difference from total brain volume, and used these volumes to account for the scaling of brain components with total brain volume [40]. We used ‘the rest of the brain’ for each individual structure instead of total brain volume when controlling for scaling effects because total brain volume includes the volume of the structure of interest. Our procedure thus ensured that the brain structure of interest was not represented on the X and Y axes simultaneously. REM and NREM sleep times were tested against each structure volume and corresponding volume of the remaining brain with multiple regression in PGLS. This allowed us to control for both phylogenetic relatedness of species and for scaling effects. We controlled for multiple testing using the false discovery rate test [FDR, 41], [42]. All tests were two-tailed with α = 0.05.

## Results

**Figure 1:**
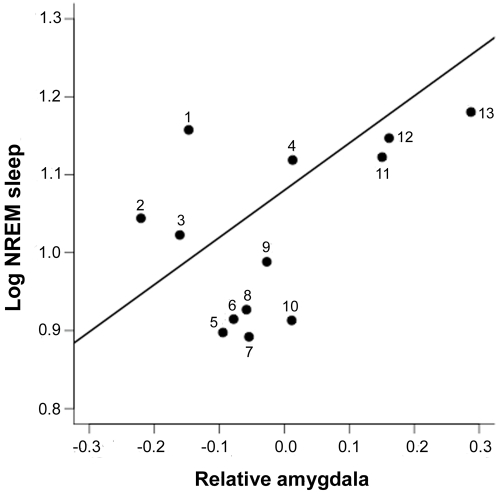
NREM sleep time and amygdala. NREM sleep durations increase with relative amygdalar volumes after accounting for scaling effects [(NREM sleep time) = 1.50+0.66 * (amygdala volumes)−0.45 * (rest of the brain); see text]. The plot shows relative amygdalar volumes, which were calculated with a phylogenetically corrected regression of amygdalar volumes on the rest of the brain, using ML in PGLS (see methods). Species number: (1) *Microcebus murinus*, (2) *Rattus norvegicus*, (3) *Nannospalax ehrenbergi*, (4) *Tupaia glis*, (5) *Callithrix jacchus*, (6) *Pan troglodytes*, (7) *Saimiri sciureus*, (8) *Papio hamadryas*, (9) *Erythrocebus patas*, (10) *Macaca mulatta*, (11) *Tenrec ecaudatus*, (12) *Erinaceus europaeus*, (13) *Aotus trivirgatus*.

After controlling for scaling effects, NREM sleep increased with amygdala volume (amygdala: t_10_ = 4.60, p = 0.001, rest of the brain: t_10_ = −4.74, p<0.001, model-R^2^ = 0.70; Figure 1) while the correlations with neocortex and hippocampus volumes were not significant (neocortex: t_11_ = −0.77, p = 0.458, rest of the brain: t_11_ = 0.65, p = 0.527, model-R^2^ = 0.06; hippocampus: t_11_ = 1.89, p = 0.086, rest of the brain: t_11_ = −1.93, p = 0.080, model-R^2^ = 0.26). We found no significant association between REM sleep durations and any of the brain structures we used (amygdala: t_10_ = 1.27, p = 0.232, rest of the brain: t_10_ = −1.39, p = 0.195, model-R^2^ = 0.16; neocortex: t_11_ = −1.71, p = 0.115, rest of the brain: t_11_ = 1.53, p = 0.153, model-R^2^ = 0.22; hippocampus: t_11_ = 0.11, p = 0.918, rest of the brain: t_11_ = −0.26, p = 0.793, model-R^2^ = 0.02). After controlling for multiple testing, NREM sleep remained significantly correlated with amygdalar volume (FDR estimated threshold of significance: α = 0.008).

## Discussion

We found that evolutionary increases in NREM sleep durations were correlated with evolutionary increases in the size of the amygdala, and this effect was independent of both scaling effects and phylogeny. We found no evidence of positive relationships between REM sleep and brain structures implicated in memory consolidation and learning. The hippocampus showed a tendency to increase with NREM sleep, although this relationship was not statistically significant (p<0.09). Sample sizes are however relatively limited in our analyses, and it would be worthwhile to re-assess this relationship once more data on sleep and brain regions have accumulated.

Our results are broadly consistent with electrophysiological, computational and neuroimaging studies that functionally link NREM slow wave sleep with information flow from amygdalar-hippocampal structures to neocortical sites during sleep [3], [22], [25], [26], [28], [43], but suggest that the amygdala is the key locus of anatomical change in sleep-regulated memory enhancement. These studies converge on a two-step model of memory consolidation that suggests an initial information transfer from the hippocampal-amygdalar complexes to neocortical sites during NREM slow wave sleep, and then a later integration of this information into existing semantic memory networks in the neocortex during REM sleep. Thus both the amygdala and the hippocampus are active during NREM sleep and only the amygdala is active during both sleep states. This may explain why we could detect a stronger link between the amygdala and NREM sleep than between the hippocampus and NREM sleep with currently available data. Insufficient comparative data exist to determine which part of the amygdala correlates most strongly with NREM sleep. The amygdala is an heterogeneous structure comprising nuclei with divergent projection systems [44]. Despite such differences, however, these nuclei have strong reciprocal connections and evolved together in a closely coordinated fashion [45].

We found no association between neocortical volumes and sleep durations. Although this result is surprising, the neocortex includes different subdivisions that undertake different functions [17] and the lack of association with sleep in our results might reflect this composite nature of the neocortex as well as small sample sizes. At present, however, sample sizes on volumes of neocortical subdivisions are not sufficient to investigate this possibility. An alternative hypothesis is that the neocortex might respond more strongly to sleep intensity than to sleep durations (see below).

Our results conflict with interpretations from some previous comparative studies of brain size and sleep durations. For example, Lesku et al. [12] found that mammals with relatively larger brains engage in relatively more REM sleep, and they concluded that this association indicates that REM sleep is important for memory consolidation, learning or other cognitive functions. In contrast, we found that NREM sleep durations, but not REM sleep durations, were linked with brain regions involved in memory processing. Although it is possible that Lesku et al. [12] identified a more generalized relationship between REM sleep and overall brain function, a subsequent study was unable to confirm this pattern when using more comparable data and different methods [11]. Thus, while our analyses provide some evidence for a memory-related function of sleep in relation to specific brain structures, there is conflicting evidence regarding the involvement of sleep in whole brain function.

Other aspects of sleep architecture are likely to be important in sleep function and evolution, particularly sleep intensity. Slow-wave activity during NREM sleep is considered to be a measure of sleep intensity and is implicated in the homeostatic regulation of sleep; sleep deprived subjects experience increases in slow-wave activity as well as the duration of subsequent sleep periods [46], [47]. Thus, memory related demands of sleep might also be met by an increase in sleep intensity. Unfortunately, there are insufficient comparative data currently available to investigate how sleep intensity may interact with sleep durations and brain structure in generating the benefits of sleep.

Our analyses were limited by the availability of data on both brain structures and sleep durations and require confirmation when larger sample sizes become available. Our study nonetheless reveals the first evidence of correlated evolution between sleep and specific brain structures, and provides support for the idea that memory consolidation may be among the ultimate functions of sleep.

## Supporting Information

Appendix S1 Sleep & Brain data
(0.01 MB XLS)
